# Cerebrovascular disease influences functional and structural network connectivity in patients with amnestic mild cognitive impairment and Alzheimer’s disease

**DOI:** 10.1186/s13195-018-0413-8

**Published:** 2018-08-18

**Authors:** Ashwati Vipin, Yng Miin Loke, Siwei Liu, Saima Hilal, Hee Youn Shim, Xin Xu, Boon Yeow Tan, Narayanaswamy Venketasubramanian, Christopher Li-Hsian Chen, Juan Zhou

**Affiliations:** 1 Centre for Cognitive Neuroscience, Neuroscience and Behavioural Disorders Program, Duke-National University of Singapore Medical School, Singapore, Singapore; 2 Department of Pharmacology, Clinical Research Centre, National University Health System, National University of Singapore, Singapore, Singapore; 3 Memory Aging and Cognition Centre, National University Health System, Singapore, Singapore; 4 Clinical Imaging Research Centre, The Agency for Science, Technology and Research and National University of Singapore, Singapore, Singapore; 5 St. Luke’s Hospital, Singapore, Singapore; 6 Raffles Neuroscience Centre, Raffles Hospital, Singapore, Singapore

**Keywords:** Neurodegeneration, Network, Functional connectivity, Structural connectivity, Alzheimer’s disease, Cerebrovascular disease, Diffusion tensor imaging

## Abstract

**Background:**

Patients with amnestic mild cognitive impairment (aMCI) and Alzheimer’s disease (AD) show functional and structural connectivity alterations in the default mode network (DMN) while cerebrovascular disease (CeVD) shows functional and structural connectivity changes in the executive control network (ECN). Such disruptions are associated with memory and executive function impairment, respectively. Concurrent AD and CeVD pathology is associated with a higher rate of cognitive decline and differential neurodegenerative patterns. Together, such findings are likely reflective of different underlying pathology in AD with and without CeVD. However, few studies have examined the effect of CeVD on network functional connectivity (task-free functional magnetic resonance imaging (fMRI)) and structural connectivity (diffusion MRI) of the DMN and ECN in aMCI and AD using a hypothesis-driven multiple seed-based approach.

**Methods:**

We examined functional and structural connectivity network changes in 39 aMCI, 50 aMCI+CeVD, 47 AD, 47 AD+CeVD, and 65 healthy controls (HCs) and their associations with cognitive impairment in the executive/attention and memory domains.

**Results:**

We demonstrate divergent DMN and ECN functional connectivity changes in CeVD and non-CeVD subjects. Compared with controls, intra-DMN hippocampal functional connectivity reductions were observed in both AD and AD+CeVD, while intra-DMN parietal and medial prefrontal-parietal functional connectivity was higher in AD+CeVD and aMCI+CeVD, but lower in AD. Intra-ECN frontal functional connectivity increases and fronto-parietal functional connectivity decreases occurred in CeVD but not non-CeVD subjects. Such functional connectivity alterations were related with cognitive impairment in a dissociative manner: intra-DMN functional connectivity changes were associated with worse cognition primarily in non-CeVD groups, while intra-ECN functional connectivity changes were associated with worse cognition primarily in CeVD groups. Additionally, CeVD and non-CeVD groups showed overlapping and distinct alterations in inter-network DMN-ECN functional connectivity depending on disease severity. In contrast to functional connectivity, CeVD groups had greater network structural connectivity damage compared with non-CeVD groups in both aMCI and AD patients. Network structural connectivity damage was associated with worse cognition.

**Conclusions:**

We demonstrate differential functional and structural network changes between aMCI and AD patients with and without CeVD through diverging and deleterious network-based degeneration underlying domain-specific cognitive impairment.

**Electronic supplementary material:**

The online version of this article (10.1186/s13195-018-0413-8) contains supplementary material, which is available to authorized users.

## Background

Alzheimer’s disease (AD) with concomitant cerebrovascular disease (CeVD) is a leading cause of age-related cognitive impairment [1]. Such a mixed pathology is not only associated with distinct neurodegenerative patterns, but also with greater cognitive decline and earlier dementia onset than AD or CeVD only [2–4].

The network-based degeneration hypothesis suggests that the disease-related spread of degeneration follows a pattern based on existing brain networks [5–8]. Emerging evidence illustrates that AD and mild cognitive impairment (MCI) are associated with functional connectivity (FC) and structural connectivity (SC) alterations in the default mode network (DMN) with associated memory impairment, while CeVD shows FC and SC changes in the executive control network (ECN) [9–13]. Recent findings from our group using single DMN/ECN seeds indicate differential neural network changes that may be reflective of different underlying pathology in subjects with and without CeVD [14]. However, most studies have used single seed-based approaches to assess FC changes in concomitant CeVD and AD. Thus, given the multiple DMN and ECN core regions and accumulative evidence on seed-dependent FC patterns, such a region-based effect of CeVD on their network connectivity in AD and amnestic MCI (aMCI) using simultaneous FC and SC approaches remains to be elucidated [6, 7, 15, 16]. Furthermore, increased vascular burden could influence cognition through network dysfunction via impaired SC [2, 17–19]. Indeed, CeVD markers have been associated with cognition in MCI [20–22]. However, the effect of CeVD on functional and structural network connectivity in AD needs further investigation, especially in aMCI [22, 23].

Given these gaps, we aimed to concurrently assess FC and SC changes within and between the DMN and ECN in aMCI and AD subjects with and without CeVD and their associations with cognitive decline using a multiple seed-based approach. We hypothesized that non-CeVD groups would show DMN FC damage underlying memory impairment while CeVD participants would show ECN FC damage underlying attention and executive function impairment. Such network divergence patterns would be less evident in SC; instead, SC disruptions are likely to be more severe in CeVD than non-CeVD.

## Methods

### Participants

Subjects were recruited from the following sites in Singapore: memory clinics at the National University Hospital, Singapore, St. Luke’s Hospital, and the community as described previously [24]. The study was approved by the National Healthcare Group Domain-Specific Review Board and conducted in accordance with the Declaration of Helsinki. Written informed consent was obtained from all participants in their preferred language prior to the start of the study. All subjects underwent medical and demographic questionnaires, physical, extensive clinical and neuropsychological assessments, neuroimaging, and diagnosis. Diagnoses of cognitive impairment and dementia were made at weekly consensus meetings where clinical features, blood investigations, psychometrics, and neuroimaging data were reviewed [25]. Detailed diagnostic criteria for aMCI, aMCI+CeVD, AD, and AD+CeVD, and inclusion/exclusion criteria are provided in Additional file 1. In brief, magnetic resonance imaging (MRI) scans were used to produce a visual rating of cortical infarcts, lacunes, and confluent white matter lesions in the brain which determined significant CeVD based on prior criteria (see Additional file 1 and our previous work [26–31]). After excluding 43 participants who either did not have MRI/diffusion tensor imaging (DTI)/functional MRI (fMRI) scans or did not pass neuroimaging data quality control, a total of 248 participants comprising 39 aMCI, 50 aMCI+CeVD, 47 AD, 47 AD+CeVD, and 65 healthy controls (HCs) were included in our study. There were no differences in disease severity or cognition between CeVD and non-CeVD groups at each AD stage (Table 1). Patients who completed cognitive assessments were included in brain cognition association analyses.

**Table 1:** Demographics and clinical characteristics of patients and control subjects

	HC (*n* = 65)	aMCI (*n* = 39)	aMCI+CeVD (*n* = 50)	AD (*n* = 47)	AD+CeVD (*n* = 47)	*p* value
Age (years)	67.3 (6.2)^b,c,d,e^	71.8 (7.9)^d^	71.4 (8.7)^d^	75.2 (7.8)	79.3 (6.1)	*p* < 0.001
Gender (M/F), *n*	29/36	22/17	29/21	17:30	15:32	*p* = 0.037
Handedness (R:L), *n*	60:5	37:2	50:0	47:0	47:0	*p* = 0.019
Ethnicity (C:M:I:O), *n*	60/2/3/0	34/1/4/0	38/8/2/2	38/6/1/2	34/9/4/0	*p* = 0.016
CDR global	0.1 (0.2)	0.4 (0.2)	0.4 (0.3)	1.1 (0.3)	1.3 (0.5)	*p* < 0.001
CDR sum of boxes	0.1 (0.2)^d,e^	0.9 (0.7)^d,e^	0.9 (0.9)^d,e^	6.2 (2.2)	6.9 (2.9)	*p* < 0.001
MMSE	27.4 (2.0)^b,c,d,e^	24.4 (3.5)^d,e^	23.6 (3.6)^d,e^	16.6 (5.2)	16.4 (4.4)	*p* < 0.001
GDS	1.57 (2.11)^e^	1.74 (2.71)	2.52 (3.02)	2.40 (2.29)	3.21 (3.16)	*p* = 0.015
WMH, cm^3^	1.97 (1.67)^c,e^	3.23 (3.11)^c,e^	14.53 (14.74)^d^	5.30 (4.84)^e^	18.11 (13.42)	*p* < 0.001
	*n* = 57	*n* = 39	*n* = 49	*n* = 37	*n* = 34	Overall ANOVA
Executive	0.81 (0.40)^b,c,d,e^	0.27 (0.70)^d,e^	0.01 (0.71)^d,e^	−0.93 (1.08)	−1.07 (0.98)	*p* < 0.001
Attention	0.63 (0.42)^b,c,d,e^	0.11 (0.56)^d,e^	−0.005 (0.52)^d,e^	−0.69 (0.91)	−0.62 (0.67)	*p* < 0.001
Language	0.81 (0.44)^b,c,d,e^	0.14 (0.55)^d,e^	0.03 (0.48)^d,e^	−0.88 (0.86)	−0.97 (0.70)	*p* < 0.001
Verbal memory	0.092 (0.47)^b,c,d,e^	−0.13 (0.64)^d,e^	−0.13 (0.59)^d,e^	−0.86 (0.51)	−0.88 (0.30)	*p* < 0.001
Visual memory	0.97 (0.37)^b,c,d,e^	−0.12 (0.49)^d,e^	−0.13 (0.39)^d,e^	−0.92 (0.50)	−0.90 (0.46)	*p* < 0.001
Visuoconstruction	0.83 (0.37)^b,c,d,e^	0.21 (0.61)^d,e^	−0.07 (0.65)^d,e^	−0.79 (0.93)	−0.91 (0.76)	*p* < 0.001
Visuomotor	0.85 (0.47)^b,c,d,e^	0.18 (0.68)^d,e^	−0.10 (0.68)^d,e^	−0.85 (0.74)	−0.89 (0.66)	*p* < 0.001

Out of the total 248 participants with imaging data, 246 participants had functional connectivity data and 247 had structural connectivity data. Out of the total 217 subjects with cognitive data, 215 had functional connectivity data and 216 had structural connectivity data

Values represent mean (SD) unless otherwise indicated

Superscript letters indicate whether group mean was significantly different compared with ^b^aMCI (B), ^c^aMCI with CeVD, ^d^Alzheimer’s disease, and ^e^Alzheimer’s disease with CeVD, based on post-hoc comparisons (*p* < 0.05) following one-way analysis of variance

Chi-square tests were carried out on sex and CDR global, while Fisher’s exact test was carried out for handedness and ethnicity covariates

*AD* Alzheimer’s disease, *aMCI* amnestic mild cognitive impairment, *ANCOVA* analysis of covariance, *C* Chinese, *CeVD* cerebrovascular disease, *CDR* Clinical Dementia Rating, *F* female, *GDS* Geriatric Depression Scale, *HC* healthy controls, *I* Indian, *L* left, *M* Malay, *M* male, *MMSE* Mini-Mental State Examination, *O* other, *R* right, *WMH* white matter hyperintensity

### Neuropsychological assessment

Diagnoses of dementia were made at weekly consensus meetings following a review of the patient’s clinical history, blood work, neuropsychological assessments, and neuroimaging data, conducted by neurologists, neuropsychologists, research nurses, and research assistants following our previous work [25, 32]. Trained research psychologists administered the following cognitive screening tests: the Mini-Mental State Examination (MMSE), the Clinical Dementia Rating Scale (CDR), the Montreal Cognitive Assessment, informant questionnaire on cognitive decline, and a detailed neuropsychological test battery locally validated for older Singaporeans [33], which assessed the following seven domains [26]: executive function (frontal assessment battery [34]; maze task [35]), attention (digit span; visual memory span [36]; auditory detection [37]), language (Boston naming test [38]; verbal fluency [39]), visuomotor speed (symbol digit modality test [40]; digit cancellation [41]), visuoconstruction (Weschler memory scale—revised visual reproduction copy task [36]; clock drawing [42]; Weschler adult intelligence scale—revised subtest of block design [43]), verbal memory (word list recall [44]; story recall), and visual memory (picture recall; visual reproduction [36]). *Z* scores were then derived for individual subtests and adapted such that a larger value reflects better performance. Summing the *z* scores of each subtest and subsequently dividing by the number of the subtests under that domain computed the overall *z* score for each individual domain. Domain-specific *z* scores were used to compute the final global cognitive composite score. The visual and verbal memory scores were combined into a composite memory score. Only subjects who completed all the tasks were included in the statistical analysis on cognition.

### Image acquisition

All subjects underwent an MRI brain scan using the 3-T Tim Trio system (Siemens, Erlangen, Germany), including a 5-min task-free fMRI scan, a T1-weighted magnetization prepared rapid gradient recalled echo sequence, a fluid attenuated inversion recovery sequence, and a DTI scan using a single-shot, echo-planar imaging sequence. White matter hyperintensity (WMH) segmentation on FLAIR images was performed using an automated procedure as described in our previous work [45, 46]. Further details are provided in Additional file 1 (Supplementary Methods).

### Image preprocessing

Task-free fMRI images were preprocessed using the Analysis of Functional Neuroimages software [47] and the FMRI Software Library (FSL) [48], following our previous protocol [24, 49]. Task-free fMRI preprocessing steps comprised the following: 1) discarding the first five images for signal stabilization and subject adaptation; 2) slice time and head motion correction; 3) despiking and grand mean scaling; 4) spatial smoothing with a 6-mm FWHM Gaussian kernel, temporal band pass filtering (0.009–0.1 Hz) and detrending (first and second order); 5) coregistering to structural MRI using boundary-based registration (BBR); 6) nonlinearly normalizing to the standard MNI space (FNIRT) via T1-weighted structural MR image; and 7) regressing out signals from white matter, cerebrospinal fluid signals, whole-brain global signal, and six head-motion parameters.

The DTI data were preprocessed by FSL following methods previously used in our studies [50]. Head movement and eddy current distortion were corrected through affine registration of diffusion-weighted images to the first b = 0 volume [51]. Data with a maximum displacement relative to the first b = 0 volume more than 3 mm were discarded. Diffusion gradients were rotated with reference to the motion parameters to improve data consistency. Individual maps were visually inspected for signal dropout and artifacts. To enable probabilistic tractography, the probabilistic distribution of diffusion parameters at each voxel was built up by Bayesian estimation of diffusion parameters (bedpostx) [52, 53].

### Network connectivity derivation

#### Region of interest (ROI) derivation

To test whether and how the DMN and ECN structural and functional network phenotype patterns in aMCI and AD with and without CeVD vary by seeding different key network regions, we employed a multiple-seed approach. We defined nine ROIs covering the DMN and ECN regions. Each spherical ROI was created with their centers determined as key nodes in the DMN and ECN by previous studies (MarsBaR package; SC: 10 mm radius; FC: 6 mm radius) [6, 8, 54]. ECN seeds included the left and right dorsolateral prefrontal cortex (lDLPFC and rDLPFC; ±45 16 45 [54]) and the left and right posterior parietal cortex (lPPC and rPPC; ±50 –50 51 [54]). DMN seeds included the left and right parahippocampal cortex (lParaHC and rParaHC; ±22 –10 –24 [8]), medial prefrontal cortex (mPFC; −16 48 44 [6]), and medial parietal regions (posterior cingulate cortex (PCC); −7 –43 33 [54]; and precuneus (PCUN); 1 –60 30 [54]). Seeds involved in the network SC analyses were placed to be centered in gray matter while at the same time sized at 10 mm to increase their chances of touching the gray and white matter boundary following previous studies [55, 56].

#### Functional connectivity (FC) derivation

Intrinsic connectivity networks using all nine seeds at the individual level were obtained using a seed-based approach following our previous work [57]. The mean time series of each spherical ROI were extracted from each participant’s preprocessed functional images. Subsequently, Pearson’s correlation was computed between each voxel’s spontaneous BOLD (blood oxygen level-dependent signal) time series and the average time series for each ROI and converted to *z* scores using Fisher’s *r*-to-*z* transformation. Group-averaged functional connectivity maps for each seed were calculated for each group, which demonstrated highly overlapping but distinctive connectivity in the DMN and ECN (Additional file 1: Figure S1).

#### Structural connectivity (SC) derivation

Each individual’s diffusion image was first coregistered to the corresponding high-resolution T1 structural image using BBR [58]. T1 structural images were nonlinearly registered to the MNI space using FSL FNIRT. The derived transformation parameters were subsequently inversed and applied on the seeds to produce seeds in the diffusion native space.

We carried out probabilistic fiber tractography on these seeds using the DTI analysis software PANDA (Additional file 1: Figure S2). A sampling of 5000 × *n* streamline fibers (5000 fibers per voxel) was carried out for each seed region with *n* number of voxels. The connectivity probability from one seed region to a given seed region was thus defined as the number of fibers passing through the given seed divided by the total number (5000 × *n*) of sampled fibers [52, 53, 59, 60]. The unidirectional connectivity probability *P*_ij_ between two seeds i and j was the weighted mean of the two individual connectivity probabilities i ➔ j and j ➔ i. Subsequently a subject-level SC matrix of the connectivity probabilities for all 36 edges between nine ROIs was created. These probabilities were then logarithmically transformed and normalized for statistical analyses.

### Statistical analyses

#### Functional connectivity group differences

Second-level analyses for each seed-based FC map were performed using Statistical Parametric Mapping (SPM12; http://www.fil.ion.ucl.ac.uk/spm/software/spm12/) software. We created an analysis of covariance (ANCOVA) model where age, sex, handedness, and ethnicity were included as covariates and groups were modeled as separate covariates. Pair-wise two-sample *t* tests were conducted to assess group differences between each of the disease groups and HCs as well as head-to-head comparisons between CeVD and non-CeVD groups. To study within-network and inter-network group differences, group-averaged network masks for the DMN and ECN were defined (see Additional file 1). Results were thresholded at a height threshold of *p* < 0.01 and a cluster-extent threshold of *p* < 0.05.

#### Structural connectivity group differences

For SC, all 36 edges between nine ROIs were classified as belonging to intra-DMN, intra-ECN, or inter-network DMN-ECN (Additional file 1: Table S1). We tested the group differences in SC of the two networks by performing edge-wise ANCOVA analysis on all 36 edges with age, sex, handedness, and ethnicity as nuisance variables. Bonferroni post-hoc pair-wise analyses were also performed on edges with significant group effect in the ANCOVA model. Results are reported at a threshold of *p* < 0.05 following correction for multiple comparisons across groups. Following this, multiple comparisons correction across 36 edges was conducted using FWE-correction at *p* < 0.0013.

#### Association between brain connectivity and cognition

To assess the association between intra- and inter-network SC/FC and cognition and the influence of CeVD on this relationship, we ran a step-wise multiple regression model across all patients (aMCI/AD) with and without CeVD separately (IBM SPSS software, version 24.0, Chicago, IL, USA). Cognitive test *z* scores for executive function, attention, verbal memory, and visual memory domains comprised the dependent variables since they represent the major deficits observed in subjects with AD and CeVD [2, 4]. We built two multiple regression models for each of the four cognitive domains (one for CeVD and one for non-CeVD) in which FC measures that showed significant group differences in AD compared with HCs or AD+CeVD compared with HCs comprised the independent variables. Age, sex, handedness, years of education, and ethnicity covariates comprised the nuisance variables in the model. The same multiple regression models were also built for SC measures and cognitive *z* scores. Beta and *p* values are reported at *p* < 0.0125, corrected for multiple comparisons across the four cognitive domains.

## Results

### Intra-network and inter-network group differences in functional connectivity

#### Intra-DMN FC group differences

**Fig. 1:**
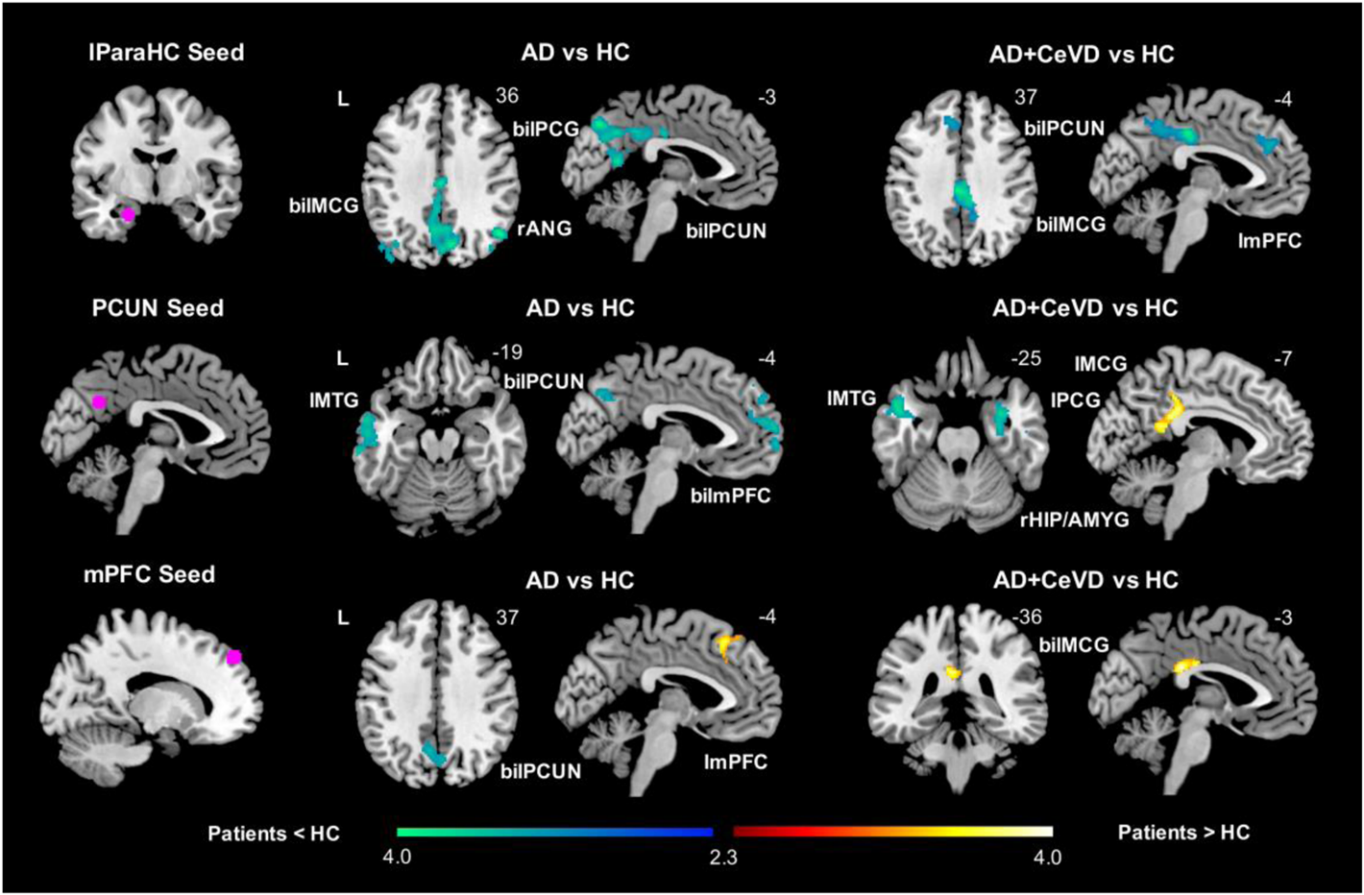
Distinct default mode network functional connectivity changes in subjects with and without cerebrovascular disease. Group functional connectivity difference maps were overlaid on the MNI template brain. The maps highlight regions showing increased (hot color) or decreased functional connectivity (cold color) in patient groups compared with HCs for the default mode network (DMN). Both AD and AD+CeVD subjects had reduced intra-DMN FC in hippocampal regions of the network for the lParaHC and PCUN seeds. Some medial prefrontal-parietal/temporal and parietal FC decreases were observed in AD subjects for the lParaHC, PCUN, and mPFC seeds, while parietal FC increases were observed in AD+CeVD subjects for the PCUN and mPFC seeds. AD subjects also showed increases in local medial prefrontal FC for the mPFC seed. AD Alzheimer’s disease, aMCI amnestic mild cognitive impairment, ANG right angular gyrus, bil bilateral, CeVD cerebrovascular disease, FC functional connectivity, HC healthy controls, HIP/AMYG hippocampus/amygdala, l left, MCG bilateral middle cingulate gyrus, MTG left middle temporal gyrus, mPFC medial prefrontal cortex, ParaHC parahippocampal cortex, PCG bilateral posterior cingulate gyrus, PCUN precuneus, r right

Seed-based voxel-wise FC ANCOVA analyses revealed largely posterior intra-DMN temporoparietal reductions for the lParaHC, PCC, and PCUN seeds in AD subjects relative to controls. AD patients had decreases in intra-DMN medial prefrontal-parietal FC but increases in anterior DMN local frontal FC (Fig. 1; Additional file 1: Table S2A). Thus, AD subjects showed lower FC in both the posterior and anterior DMN regions compared with controls but higher FC for only the mPFC seed. aMCI subjects did not show any intra-DMN FC alterations compared with controls.

Subjects with CeVD showed overlapping but distinct intra-DMN FC changes. Specifically, AD+CeVD subjects also showed reductions in posterior DMN FC for the bilateral ParaHC seeds and PCC-temporal and PCUN-temporal FC. However, in contrast to non-CeVD, CeVD groups showed increased intra-DMN FC for the mPFC, PCC, and PCUN seeds. Specifically, local intra-DMN parietal FC exhibited increases in both AD+CeVD and aMCI+CeVD subjects for the PCC and PCUN seeds. Additionally, for the mPFC seed, AD+CeVD and aMCI+CeVD showed increased medial prefrontal-parietal FC (Fig. 1; Additional file 1: Figure S3 and Table S2A). Overall, increases in intra-DMN FC were predominantly observed in CeVD subjects.

#### Intra-ECN FC group differences

**Fig. 2:**
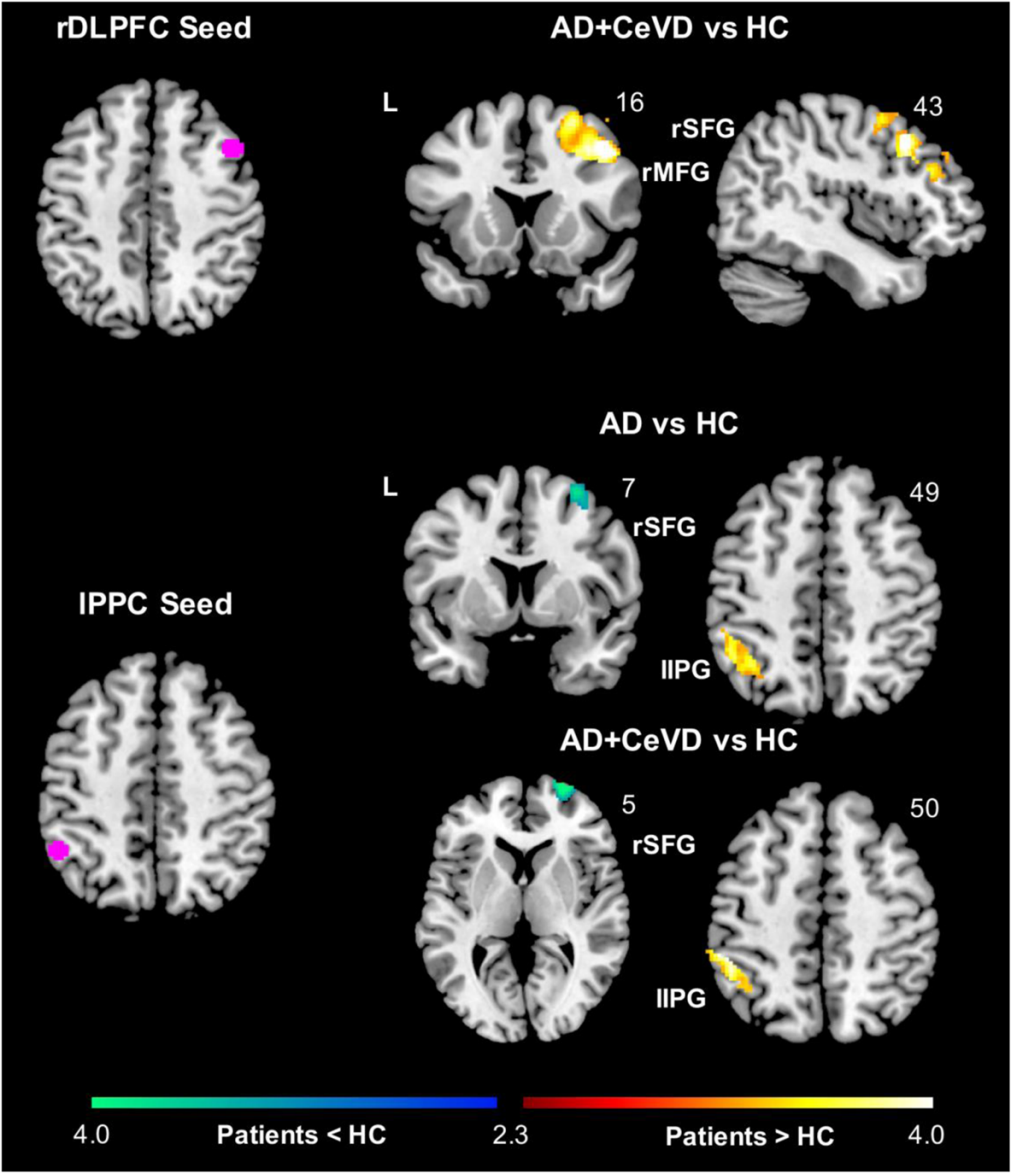
Distinct executive control network functional connectivity changes in subjects with and without cerebrovascular disease. Group functional connectivity difference maps were overlaid on the MNI template brain. The maps highlight regions showing increased (hot color) or decreased functional connectivity (cold color) in patient groups compared with HCs for the executive control network (ECN). AD+CeVD subjects showed increased intra-ECN frontal FC for the rDLPFC seed and parietal FC for the lPPC seed while AD subjects showed higher parietal FC compared with HCs for the lPPC seed. Frontoparietal FC was reduced in AD and AD+CeVD subjects compared with HCs for the lPPC seed. AD Alzheimer’s disease, aMCI amnestic mild cognitive impairment, ANG right angular gyrus, CeVD cerebrovascular disease, FC functional connectivity, HC healthy controls, IPG inferior parietal gyrus, l left, lPPC left posterior parietal cortex, MFG middle frontal gyrus, rDLPFC right dorsolateral prefrontal cortex, r right, SFG superior frontal gyrus

Overall, intra-ECN FC was affected to the largest extent in CeVD subjects. Compared with controls, local intra-ECN FC showed increases across all four ECN seeds (i.e., lDLPFC, rDLPFC, lPPC, and rPPC), predominantly in AD+CeVD. Specifically, AD+CeVD subjects showed increases in local frontal FC for the lDLPFC and rDLPFC seeds and increases in local parietal FC for the lPPC and rPPC seeds. On the other hand, aMCI+CeVD subjects showed both increases and decreases in frontal FC for the rDLPFC seed. No other DLPFC-related FC reductions were observed. Such increases in frontal FC for the DLPFC seeds were associated with higher WMH volume (Additional file 1: Supplementary Results 2.5 and Figure S5). Additionally, AD+CeVD and aMCI+CeVD showed decreased frontoparietal FC for the lPPC seed while AD+CeVD subjects showed decreased frontal FC for the rPPC seed (Fig. 2; Additional file 1: Figure S4 and Table S2B).

In contrast, AD subjects did not show any intra-ECN DLPFC-related FC changes. However, for both the lPPC and rPPC seeds, AD subjects showed decreased frontoparietal FC and increased local parietal FC for the lPPC seed (Fig. 2; Additional file 1: Table S2B). Similar to intra-DMN, no intra-ECN FC alterations were observed in aMCI subjects relative to controls.

#### Inter-network FC group differences

Inter-network frontotemporal FC and frontoparietal FC was reduced in AD subjects. In contrast, AD+CeVD subjects showed reductions in frontotemporal, temporoparietal, and frontoparietal FC, but also increases in medial prefrontal-frontal, parietal, and frontoparietal FC (Additional file 1: Table S2C). Further details are provided in Additional file 1 (Supplementary Results).

#### Group differences in intra- and inter-network FC between CeVD and non-CeVD groups

Head-to-head comparisons between CeVD and non-CeVD groups were consistent with intra-DMN and intra-ECN FC changes compared with controls and are detailed in Additional file 1 (Supplementary Results and Table S3). Inter-network DMN-ECN FC showed disease stage-dependent divergent alterations between CeVD and non-CeVD subjects (Additional file 1: Table S3C). Further details are provided in Additional file 1 (Supplementary Results).

Although none of our participants had a clinical diagnosis of depression, to control for the potential influence of mild depressive symptoms on FC we repeated the same analyses on group differences in intra- and inter-network FC after controlling for Geriatric Depression Scale scores. All the main findings remained the same (Additional file 1: Tables S2 and S3).

### CeVD groups had more early and severe structural connectivity disruptions than non-CeVD groups

**Fig. 3:**
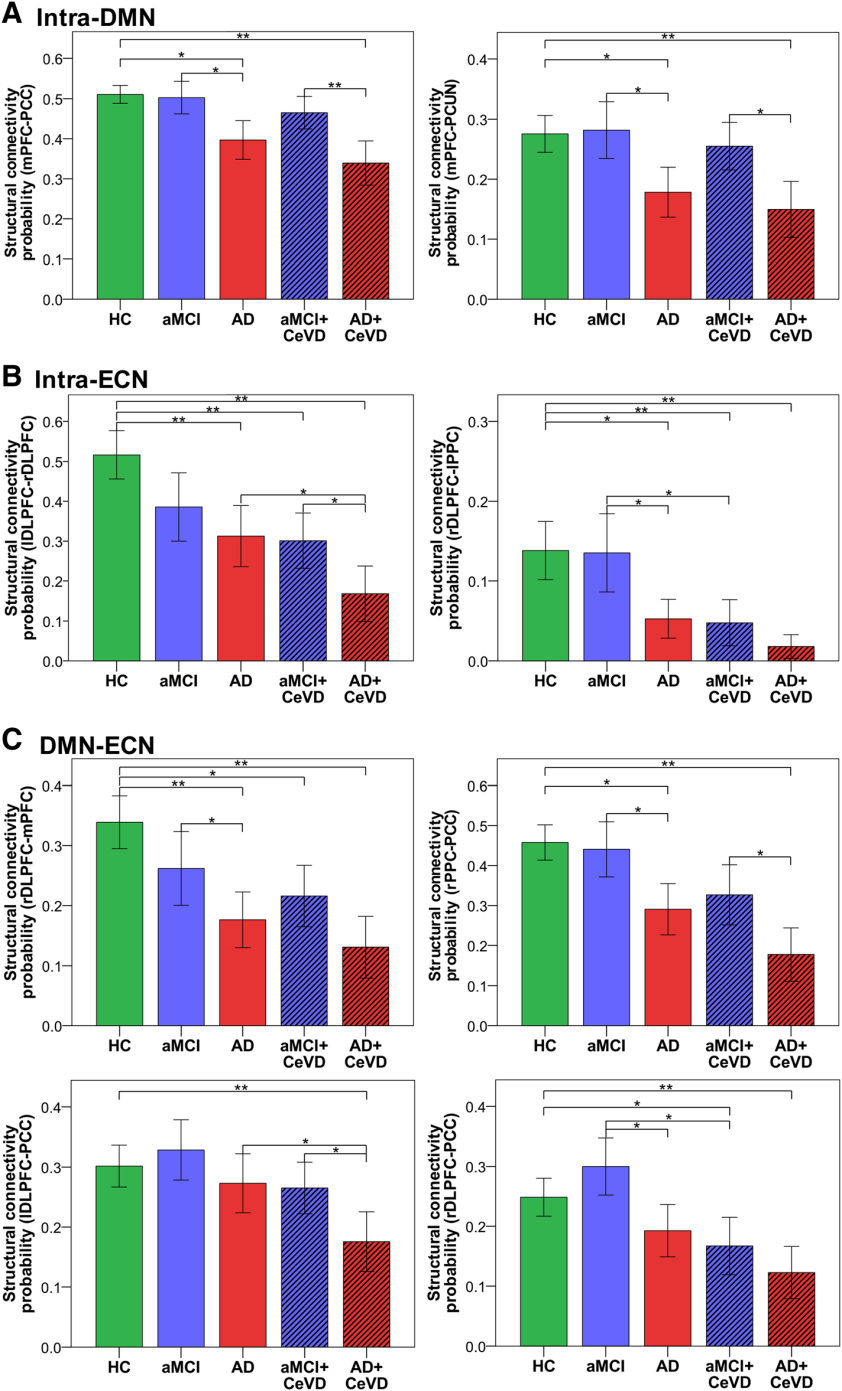
Patients with cerebrovascular disease had more severe and early structural connectivity disruptions. Representative edges showing group differences in structural connectivity. **a** Both AD and AD+CeVD subjects had reduced intra-DMN SC compared with both controls and aMCI+CeVD but AD+CeVD subjects had more widespread damage (Table 2). **b** Intra-ECN SC was reduced in AD+CeVD compared with controls and aMCI+CeVD as well as AD participants (left). Additionally, subjects with aMCI+CeVD also had reduced intra-ECN SC compared with controls and aMCI only subjects (right). **c** Inter-network SC showed reduced connectivity in AD+CeVD compared with controls and aMCI+CeVD and in AD subjects compared with controls and aMCI. Additionally, the bottom two panels illustrate SC disruption between AD+CeVD and AD as well as aMCI+CeVD and aMCI participants. Pairwise comparisons were corrected for multiple comparisons. *p < 0.05, **p < 0.001. AD Alzheimer’s disease, aMCI amnestic mild cognitive impairment, CeVD cerebrovascular disease, DMN default mode network, ECN executive control network, HC healthy controls, lDLFPC left dorsolateral prefrontal cortex, lPPC left posterior parietal cortex, mPFC medial prefrontal cortex, PCC posterior cingulate cortex, PCUN precuneus, rDLFPC right dorsolateral prefrontal cortex, rPPC right posterior parietal cortex, SC structural connectivity

Intra- and inter-network SC progressively worsened from controls to aMCI and AD (Additional file 1: Table S3). The largest reduction in SC was observed in AD+CeVD subjects. Both AD subjects with and without CeVD showed intra-DMN temporal, frontoparietal, and temporoparietal (Fig. 3a), intra-ECN frontal, frontoparietal, and parietal (Fig. 3b), and inter-network frontal, parietal, frontoparietal, and temporoparietal SC disruptions (Fig. 3c). However, SC reductions were more widespread in CeVD than in non-CeVD groups.

At the aMCI stage, aMCI subjects without CeVD showed no SC changes. In contrast, aMCI+CeVD participants had intra-ECN (rDLPFC-lPPC; lDLPFC-rDLPFC) and ECN-DMN (rDLPFC-mPFC; rDLPFC-PCC) SC reductions compared with controls (Fig. 3; Additional file 1: Table S3). Frontoparietal intra-ECN (rDLPFC-lPPC; lDLPFC-rDLPFC) and inter-network DMN-ECN (rDLPFC-PCC; lDLPFC-PCC) connections also showed reduced SC in aMCI+CeVD and AD+CeVD subjects compared with non-CeVD aMCI and AD subjects, respectively (Table 2). Such a pattern of SC deterioration remained after controlling for log-transformed WMH volume (Additional file 1: Table S4).

**Table 2:** Group differences in structural connectivity

	Without CeVD			With CeVD				
	HC > aMCI	HC > AD	aMCI > AD	HC > aMCI+CeVD	HC > AD+CeVD	aMCI+CeVD > AD+CeVD	aMCI > aMCI+CeVD	AD > AD+CeVD
Intra-DMN								
lParaHC-rParaHC	–	–	0.019	–	0.032	–	–	–
lParaHC-mPFC	–	–	–	–	0.002	–	–	–
lParaHC-PCUN	–	–	–	–	–	0.010	–	–
rParaHC-PCC	–	–	–	–	0.030	0.019	–	–
mPFC-PCC	–	0.002	0.041	–	< 0.001*	< 0.001*	–	–
mPFC-PCUN	–	0.003	0.011	–	< 0.001*	0.003	–	–
Intra-ECN								
lDLPFC-rDLPFC	–	0.001*	–	0.001*	< 0.001*	0.025	–	0.039
lDLPFC-rPPC	–	< 0.001*	0.042	–	< 0.001*	–	0.004	–
rDLPFC-lPPC	–	0.004	0.008	0.001*	< 0.001*	–	–	–
rDLPFC-rPPC	–	–	–	–	0.027	–	–	–
lPPC-rPPC	–	0.001*	0.010	–	0.001*	–	–	–
DMN-ECN								
lDLPFC-lParaHC	–	–	–	–	0.001*	–	–	–
lDLPFC-PCC	–	–	–	–	< 0.001*	0.008	–	0.007
lDLPFC-PCUN	–	–	–	–	0.021	–	–	–
rDLPFC-mPFC	–	< 0.001*	0.028	0.004	< 0.001*	–	–	–
rDLPFC-PCC	–	–	0.004	0.037	< 0.001*	–	0.001*	–
rDLPFC-PCUN	–	–	–	–	0.007	–	–	–
lPPC-rParaHC	–	–	0.022	–	0.047	–	–	–
lPPC-mPFC	–	–	–	–	–	0.043	–	–
lPPC-PCC	–	–	–	–	< 0.001*	–	–	–
rPPC-rParaHC	–	–	–	–	0.001*	0.023	–	–
rPPC-mPFC	–	–	–	–	< 0.001*	–	–	–
rPPC-PCC	–	0.021	0.010	–	< 0.001*	0.023	–	–

Structural connectivity probabilities were logarithmically transformed and normalized for statistical analyses. There were no group differences in SC between HCs and aMCI without CeVD subjects. AD+CeVD subjects had the largest reduction in SC at both the intra- and inter-network level compared with both HCs and aMCI with and without CeVD. We detected a reduction in SC in aMCI+CeVD subjects compared with both controls and aMCI subjects. AD+CeVD and aMCI+CeVD showed intra-ECN and inter-network SC reductions compared with AD and aMCI without CeVD subjects, respectively

Each cell represents the *p* value for significant pair-wise comparisons in SC at a threshold of *p* < 0.05 following correction for multiple comparisons across groups

*AD* Alzheimer’s disease, *aMCI* amnestic mild cognitive impairment, *CeVD* cerebrovascular disease, *DMN* default mode network, *ECN* executive control network, *HC* healthy controls, *lDLFPC* left dorsolateral prefrontal cortex, *lParaHC* left parahippocampus, *lPCC* left posterior cingulate cortex, *lPPC* left posterior parietal cortex, *mPFC* medial prefrontal cortex, *rDLFPC* right dorsolateral prefrontal cortex, *rParaHC* right parahippocampus, *rPCUN* right precuneus, *rPPC* right posterior parietal cortex, *SC* structural connectivity

*Comparisons that passed multiple comparisons correction across the 36 edges at *p* < 0.0013

### CeVD and non-CeVD groups feature differential structural and functional dysconnectivity underlying memory and executive functioning deficits

**Fig. 4:**
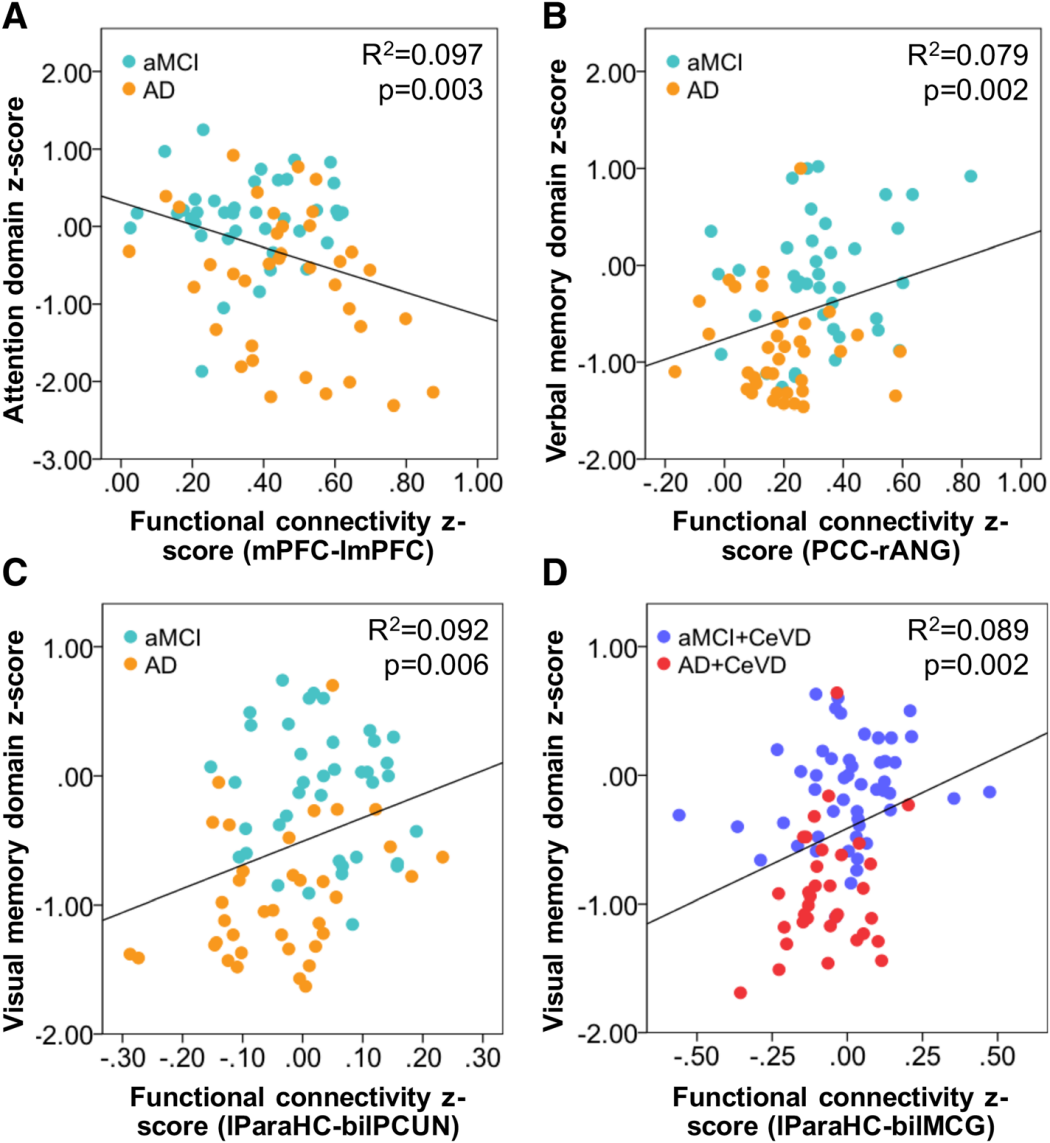
Intra-DMN functional connectivity relates to memory and executive/attention function in subjects without cerebrovascular disease and memory in subjects with cerebrovascular disease. Representative regions showing associations between intra-DMN functional connectivity and cognition. In aMCI and AD subjects without CeVD, **a** FC between mPFC and left superior mid frontal gyrus was negatively associated attention function while **b** higher parietal FC between PCC and right angular gyrus was associated with higher verbal memory and **c** higher temporoparietal FC between lParaHC and bilateral PCUN was associated with better visual memory. In AD and aMCI subjects with CeVD, **d** higher temporoparietal FC between the lParaHC and bilateral middle cingulate gyrus was positively associated with visual memory. All FC cognitions shown pass the multiple comparisons correction for number of cognitive domains at *p* < 0.0125. AD Alzheimer’s disease, aMCI amnestic mild cognitive impairment, ANG right angular gyrus, bil bilateral, CeVD cerebrovascular disease, DLFPC dorsolateral prefrontal cortex, DMN default mode network, FC functional connectivity, l left, MCG middle cingulate gyrus, mPFC medial prefrontal cortex, ParaHC parahippocampal cortex, PCC posterior cingulate cortex, PCUN precuneus, r right, SC structural connectivity

Intra-DMN FC associations with cognition in both memory and non-memory domains were primarily observed in AD and aMCI subjects without CeVD (Additional file 1: Table S5). Specifically, higher frontal intra-DMN FC (mPFC-left superior medial frontal gyrus) was associated with worse executive (*p* = 0.003; *r* = 0.28), attention (*p* = 0.003; *r* = 0.31) (Fig. 4a), and visual memory (*p* = 0.007; *r* = 0.28). Furthermore, higher temporoparietal (PCC-lParaHC; lParaHC-bilateral precuneus) FC was associated with better executive function (*p* = 0.009; *r* = 0.36) and visual memory (*p* = 0.006; *r* = 0.30) (Fig. 4c), respectively, and higher parietal (PCC-right angular gyrus) FC was associated with better verbal memory (*p* = 0.002; *r* = 0.28) (Fig. 4b). In AD+CeVD and aMCI+CeVD subjects, only higher temporoparietal (lParaHC-bilateral middle cingulum) FC was associated with better verbal (*p* = 0.005; *r* = 0.30) and visual memory (*p* = 0.002; *r* = 0.30) (Fig. 4d).

**Fig. 5:**
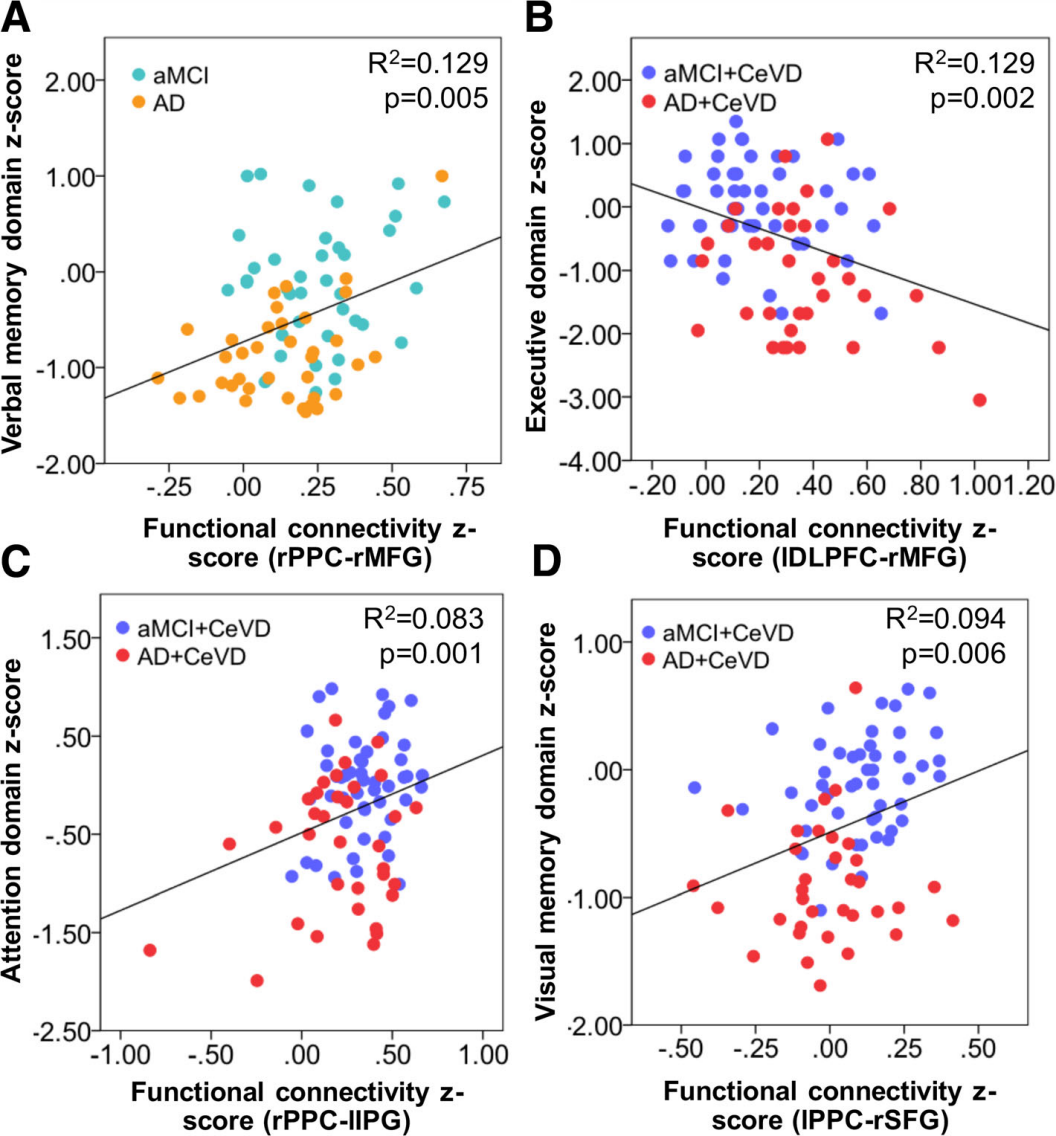
Intra-ECN functional connectivity relates to memory and executive/attention in subjects with cerebrovascular disease and to memory in subjects without cerebrovascular disease. Representative regions showing associations between intra-ECN functional connectivity and cognition. **a** In AD and aMCI subjects without CeVD, higher frontoparietal FC between rPPC and right middle superior frontal gyrus was associated with better verbal memory. In AD and aMCI subjects with CeVD, **b** higher frontal FC between lDLPFC and right middle frontal gyrus was associated with worse executive function, **c** higher parietal FC between rPPC and left inferior parietal gyrus was associated with better attention function, and **d** higher frontoparietal FC between lPPC and right superior frontal gyrus was associated with better visual memory. All FC cognitions shown pass the multiple comparisons correction for number of cognitive domains at *p* < 0.0125. AD Alzheimer’s disease, aMCI amnestic mild cognitive impairment, CeVD cerebrovascular disease, DLPFC dorsolateral prefrontal cortex, DMN default mode network, ECN executive control network, FC functional connectivity, IPG inferior parietal gyrus, l left, MFG middle frontal gyrus, PPC posterior parietal cortex, r right, SFG superior frontal gyrus,

In contrast, intra-ECN FC associations with cognition in both memory and non-memory domains were primarily observed in AD and aMCI subjects with CeVD (Additional file 1: Table S5). Specifically, higher frontal (lDLPFC-right middle frontal gyrus) FC was associated with worse executive function (*p* = 0.002; *r* = 0.35) (Fig. 5b), attention (*p* = 0.003; *r* = 0.33), and visual memory (*p* = 0.002; *r* = 0.34). Higher parietal FC (rPPC-left inferior parietal gyrus) was associated with better attention (*p* = 0.001; *r* = 0.29) (Fig. 5c) while higher frontoparietal (lPPC-right superior frontal gyrus) FC was associated with better visual memory (*p* = 0.006; *r* = 0.31) (Fig. 5d). Only higher frontoparietal (rPPC-right middle superior frontal gyrus) FC was associated with better verbal memory (*p* = 0.005; *r* = 0.36) in AD and aMCI subjects without CeVD (Fig. 5a). Additionally, there were no associations between intra-DMN FC and non-memory function in CeVD groups and between intra-ECN FC and non-memory function in non-CeVD groups.

SC deterioration was associated with cognitive impairment in a similar manner between CeVD and non-CeVD groups (Additional file 1: Table S6). These findings remained significant after controlling for total WMH volume. Further details are provided in Additional file 1 (Supplementary Results).

## Discussion

A hypothesis-driven multiple seed-based approach and combination of functional and structural connectivity analyses were used to assess the effect of CeVD on DMN and ECN connectivity in aMCI and AD patients. We demonstrated region-specific FC changes in AD patients with and without CeVD, which related to cognitive impairment. Both AD and AD+CeVD subjects showed reductions in hippocampal FC within the DMN. However, parietal and medial prefrontal-parietal DMN FC was increased in CeVD groups but decreased in AD subjects. As predicted, intra-ECN alterations in frontal and frontoparietal FC were observed most extensively in CeVD subjects. Notably, aMCI+CeVD subjects exhibited similar intra-network FC changes to AD+CeVD, while aMCI subjects did not show any intra-network FC changes compared with HCs. Inter-network FC reductions were observed in AD and AD+CeVD subjects, while aMCI and aMCI+CeVD subjects primarily showed increases when compared with controls. Direct comparisons between CeVD and non-CeVD groups revealed disease severity-dependent alterations in inter-network FC with decreased DMN-ECN FC in aMCI+CeVD compared with aMCI but increased DMN-ECN FC in AD+CeVD compared with AD. Moreover, intra-DMN FC changes were associated with cognitive impairment primarily in non-CeVD groups while ECN-related FC changes were associated with cognitive impairment primarily in CeVD groups. Additionally, CeVD groups had greater SC damage within and between the two networks compared with non-CeVD groups at both aMCI and AD stages. Similar to our FC findings, aMCI with CeVD but not those without CeVD had SC declines. This study suggests that subjects with CeVD show distinct network FC phenotypes and severe SC deterioration in the brain which underlie cognitive impairment.

The DMN is important for cognitive functions such as episodic memory and has been widely implicated in AD [5, 7]. Our non-CeVD and CeVD AD patients showed extensive intra-DMN FC alterations. However, posterior DMN FC alterations involving the posterior cingulate, precuneus, and hippocampus seeds were dominant in AD subjects, as observed previously [12, 61]. These regions have been shown to comprise the core DMN as well as being involved in early amyloid deposition and associations with autobiographical and episodic memory [5, 62]. In support of such findings, intra-DMN FC and cognition associations were primarily observed in non-CeVD groups in our study. Additionally, AD subjects showed increases in frontal FC which were negatively associated with cognition, thus indicating that such increases were derogatory in nature [63, 64]. On the other hand, intra-DMN medial prefrontal-parietal FC was decreased in AD subjects but increased in both aMCI and AD with CeVD. Such a divergence in FC changes between CeVD and non-CeVD subjects could possibly be due to disruption of frontal pathways in the presence of vascular disease [65]. Indeed, associations between intra-ECN frontal FC increase and frontal SC decrease were found in AD groups in our study (Additional file 1: Supplementary Results section 2.6). Thus, while AD subjects both with and without CeVD showed similar involvement of hippocampal FC, medial prefrontal-parietal FC was instead differentially targeted in CeVD and non-CeVD, likely indicative of differential subnetwork FC alterations in the presence of CeVD.

Widespread intra-ECN FC alterations including increases in frontal FC were observed in AD+CeVD subjects, possibly reflecting greater influences on ECN connectivity in CeVD [11, 14]. We also found associations between higher frontal ECN FC and higher WMH volume in both aMCI and AD groups (with and without CeVD) and postulate that such increases in ECN FC could be representative of CeVD abnormalities in the brain (Additional file 1: Figure S5). Additionally, such increases in frontal FC were associated with worse executive, attention, and memory function in subjects with CeVD, indicating a derogatory influence. Parietal ECN FC was reduced in CeVD subjects and was associated with worse attention function. Indeed, associations between markers of CeVD (WMH and lacunes) and executive/attention function have been demonstrated [2, 11, 18]. Moreover, task-based fMRI studies in the healthy elderly with CeVD and resting-state fMRI studies in vascular cognitive impairment have shown alterations in ECN connectivity [66]. Importantly, associations between ECN FC and cognition were primarily observed in subjects with CeVD in our study. Thus, in line with previous studies, our findings further lend evidence to the influence of concomitant AD and CeVD on network FC and cognition.

Furthermore, findings from our group and others show inter-network segregation as being consistently affected in AD patients and point towards its role in cognition [49, 67]. Interestingly, we observed lower DMN-ECN frontoparietal FC in aMCI+CeVD compared with aMCI, but higher frontoparietal FC in AD+CeVD compared with AD subjects. Such differential inter-network FC changes at the aMCI and AD stages likely provide some evidence for stage-dependent alterations in network segregation in the presence of CeVD. While reductions in aMCI+CeVD inter-network FC possibly reflect a compensatory mechanism in the presence of CeVD, increased inter-network FC with disease progression to AD+CeVD might reflect a breakdown in inter-network segregation possibly due to CeVD-related neuronal loss and degradation of white matter networks [68].

Prior FC studies have demonstrated inconsistent findings regarding disruptions in MCI [12, 21]. For example, whole-brain FC studies have shown both FC decreases and increases in parietal and temporal regions, reflecting a concurrent state of impairment as well as compensation [61, 63]. In this study, intra-network FC alterations were observed in aMCI+CeVD subjects when compared with controls, which largely mirrored alterations observed in AD+CeVD subjects [14]. Interestingly, no intra-network FC alterations occurred in the aMCI-only subjects. This indicates that aMCI+CeVD subjects appear to be further along the disease spectrum than non-CeVD aMCI subjects. We speculate that the absence of FC changes might also reflect a possible compensatory mechanism accompanied by network reorganization in aMCI, which may breakdown in the presence of CeVD [61, 63]. Further studies integrating task-based and task-free FC methods are required to study how CeVD influences whole-brain network topology and its relationship with cognitive impairment in aMCI.

In concordance with our FC patterns of large-scale alterations, our findings indicated that, overall, CeVD groups showed more widespread SC changes compared with non-CeVD groups [17]. Importantly, SC disruptions in CeVD groups occurred primarily along intra-ECN (i.e., frontal or fronto-parietal connections such as between the DLPFC and PPC). Our observations are supported by prior studies showing decreased frontal and parietal nodal efficiency in CeVD and its mediating effect on frontal lobe structure and cognition [13]. Direct comparisons between aMCI and AD subjects with and without CeVD also highlight greater intra-ECN SC damage. Crucially, we found early intra-ECN and inter-network SC damage with sparing of intra-DMN fibers in aMCI+CeVD subjects, in agreement with prior studies [13, 17]. As observed in our seed-based FC analyses, these differences in SC were not observed in the non-CeVD aMCI subjects. Such findings indicate an ECN-specific structural and additive influence of CeVD that likely begins in aMCI. Additionally, and unlike FC, there was no dissociation in the SC-cognition relationship between CeVD and non-CeVD groups. Performance on both memory and executive/attention domains was associated with intra-network SC in both AD and aMCI subjects with and without CeVD, indicating that white matter damage might lead to deficiencies in both memory and executive/attention domains regardless of CeVD status [20, 21]. Our SC findings reflect that CeVD may be associated with greater white matter degeneration and lend evidence to the additive hypothesis regarding the influence of CeVD when there is concomitant AD [3, 19].

Our study has some limitations. As a hypothesis-driven seed-based approach was chosen to compare SC and FC in the two networks of interest, these findings may be affected by inter-subject anatomical variability. A relatively large proportion of the CeVD subjects in our study had infarcts in the frontal regions (Additional file 1: Table S8), which may bias the ECN functional connectivity estimation and associations with cognition. Additionally, although groups were not age-matched and disease duration was not available, age differences were accounted for in all analyses and disease severity was matched between CeVD and non-CeVD groups at the aMCI and AD stages, respectively. It has also been suggested that probabilistic fiber tracking can be influenced by the presence of WMH in the brain [69]. While we did control for WMH volume in our structural connectivity statistical analyses, WMH may still confound the fiber tracking results, especially in the crossing-fiber regions. Furthermore, out of the 248 subjects included in our study, only 45 (9 HC, 16 aMCI, 14 aMCI+CeVD, and 6 AD) had amyloid imaging data. Thus, we are unable to assess how the heterogeneity in the etiology of the patient groups, especially at the aMCI stage, would have influenced our findings.

## Conclusions

In summary, we demonstrate distinct network FC phenotypes underlying cognitive impairment in patients with and without CeVD and provide important evidence for the influence of CeVD on early structural network disruptions. Our findings highlight the value of concurrent SC and FC neuroimaging assays to reveal early changes and distinct pathology in mixed cerebrovascular and neurodegenerative disorders. Future longitudinal studies are required to investigate the influence of CeVD on disease progression trajectory and network changes in preclinical AD.

## Additional file

Additional file 1: Supplementary methods, results, tables, and figures. (DOCX 37000 kb)

## Abbreviations

AD: Alzheimer’s disease
aMCI: Amnestic mild cognitive impairment
ANCOVA: Analysis of covariance
BBR: Boundary-based registration
CeVD: Cerebrovascular disease
DMN: Default mode network
DTI: Diffusion tensor imaging
ECN: Executive control network
FC: Functional connectivity
fMRI: Functional magnetic resonance imaging
FSL: FMRI Software Library
HC: Healthy controls
lDLPFC: Left dorsolateral prefrontal cortex
lParaHC: Left parahippocampal cortex
lPPC: Left posterior parietal cortex
MCI: Mild cognitive impairment
mPFC: Medial prefrontal cortex
MRI: Magnetic resonance imaging
PCC: Posterior cingulate cortex
PCUN: Precuneus
rDLPFC: Right dorsolateral prefrontal cortex
ROI: Region of interest
rParaHC: Right parahippocampal cortex
rPPC: Right posterior parietal cortex
SC: Structural connectivity
WMH: White matter hyperintensity
