# Supplementary material for: Cerebrovascular disease influences functional and structural network connectivity in patients with amnestic mild cognitive impairment and Alzheimer’s disease
Source: Alzheimers Res Ther. 2018 Aug 18;10:82. doi: 10.1186/s13195-018-0413-8 (PMC6098837; doi:10.1186/s13195-018-0413-8)
Supplement: Supplementary file 1 — Supplementary methods, results, tables, and figures. (DOCX 37000 kb) [file 13195_2018_413_MOESM1_ESM.docx]

**Additional file**

**1. Supplementary Methods:**

**1.1 Subjects diagnosis and inclusion/exclusion criteria**

Diagnoses were consistent with the Diagnostic and Statistical Manual of Mental Disorders (DSM)-IV criteria. Computed tomography (CT), magnetic resonance imaging (MRI), and magnetic resonance angiography were reviewed as part of the diagnostic process. Etiological diagnoses were made according to the National Institute of Neurological and Communicative Disorders and Stroke and the Alzheimer’s disease (AD) and Related Disorders Association guidelines for AD. Briefly, AD patients were classified based on gradual and slow onset of memory problems, impairment in objective neuropsychological assessment, and loss in activities of daily living. Neuroimaging evidence for significant cerebrovascular disease was assessed using the following criteria: (1) cortical infarcts; (2) two or more lacunes; and (3) confluent white matter (WM) lesions in two brain regions (Age Related WM Change scale score ≥ 8) [[1](#_ENREF_1)]. We performed the inter-rater agreement validation on the memory clinical samples used in the current study: the inter-rater agreement for infarcts was good (kappa, k=0.79) and the inter-rater reliability for white matter hyperintensity was excellent (kappa, k=0.85) in our sample. For the healthy controls (HC), we ensured that the participants had no impairment in the seven domains assessed via extensive neuropsychological tests, and their Mini-Mental State Examination (MMSE) scores were greater than or equal to 26 [[2](#_ENREF_2), [3](#_ENREF_3)].

Participants were excluded from this study if they were/had: (1) hypoxic/ anoxic, hypotensive, hypertensive, uremic or hepatic encephalopathy; (2) traumatic, nutritional or toxic disorders that affect the central nervous system (CNS); (3) current substance use disorders (e.g., alcohol, barbiturates, opiates, amphetamines, phencyclidine, and/or cocaine as defined by the DSM-IV) or previous substance use disorders that affect the CNS; (4) intracerebral haemorrhages that may cause cognitive impairments; (5) cranial arteritis, CNS inflammatory vasculitides, or Moyamoya disease; (6) CNS infections, including syphilis, tuberculosis, fungi, rickettsiae, bacterium, viral encephalitis, Creutzfeld-Jacob disease or other CNS infections; (7) space-occupying intracranial mass lesions; (8) obstructive or normal pressure hydrocephalus; (9) difficulty controlling epilepsy, which may cause cognitive impairments; or (10) schizophrenia or bipolar disorder.

**1.2 Image Acquisition**

Imaging parameters are provided as following. The task-free fMRI sequence comprised a T2*-weighted echo planar sequence with repetition time = 2300 ms, echo time = 25 ms, flip angle = 90 degrees, field of view = 192 x 192 mm^2^, voxel size = 3.0 mm isotropic, slice thickness = 3 mm, 48 axial slices, interleaved collection); the T1-weighted Magnetization Prepared Rapid Gradient Recalled Echo (MPRAGE) sequence comprised repetition time = 2300 ms, echo time = 1.9 ms, inversion time = 900 ms, flip angle = 9°, 192 sagittal slices, matrix size = 256 x 256, FOV = 256 x 256 mm^2^, voxel size = 1.0 x 1.0 x 1.0 mm^3^, bandwidth = 240 Hz/pixel; the Fluid Attenuated Inversion Recovery (FLAIR) sequence comprised repetition time = 9000 ms, echo time = 82 ms, inversion time = 2500 ms, flip angle = 180°, 48 transversal slices, matrix size = 256 x 232, voxel size = 1.0 x 1.0 x 3.0 mm^3^; diffusion tensor imaging (DTI) scan comprised a single-shot, echo-planar imaging (EPI) sequence (61 non-collinear diffusion gradient directions at b = 1150 seconds/mm^2^, seven volumes of b = 0 seconds/mm^2^, TR/TE = 6800/85 ms, FOV = 256 x 256 mm^2^, matrix = 84 x 84, voxel size = 3 mm^3^, 48 contiguous slices and slice thickness = 3.0 mm.

**1.3 White matter hyperintensity quantification**

The WMH quantification steps included: 1) registration of the FLAIR image to T1-weighted structural MRI for each individual via linear transformation based on normalized mutual information; 2) segmentation of grey matter (GM), WM, and cerebral spinal fluid (CSF) from the T1-weighted structural MR images using the Statistical Parametric Mapping (SPM8) software (<http://www.fil.ion.ucl.ac.uk/spm/)>; 3) applying this segmentation on the FLAIR image and removal of non-brain regions; 4) determination of the modal pixel intensity within the masked FLAIR image; and 5) performing a threshold-based segmentation by identifying all voxels 1.45 times or larger than the modal pixel intensity.

**1.4 Derivation of group-averaged network maps**

We performed a one-sample t-test on z-score maps of healthy control subjects for each DMN and ECN ROI to create their respective network maps (FWE-corrected p<0.001, voxel threshold = 1000 voxels). Subsequently, maps obtained for all DMN ROIs were combined i.e. lParaHC map ∪ rParaHC map ∪ mPFC map ∪ PCC map ∪ PCUN map, to create a group-averaged map for the DMN and the maps obtained for all ECN ROIs were similarly combined i.e. lDLPFC ∪ rDLPFC ∪ lPPC ∪ rPPC to create a group-averaged network map for the ECN.

**1.5 Structural connectivity derivation quality control**

As part of our regular pipeline for DTI analyses, we first performed motion quality check (based on absolute motion parameters as described in main Methods section 2.4) and visual quality check of the resulting fractional anisotropy and mean diffusivity maps in native space derived from the DTI data. Our team also carried out visual quality checks on T1 to standard space normalization and T1 to DTI registration results by overlaying the two images. Lastly, we manually checked the registered ROIs in native space to ensure its consistent location and proper shape for effective probabilistic fiber tracking across subjects. We excluded subjects failing any of these three quality control steps.

**2 Supplementary Results**

**2.1 Inter-network FC group differences between patient groups and healthy controls**

Inter-network frontotemporal FC was reduced in AD subjects compared to controls for lParaHC and rParaHC seeds. Temporoparietal and frontotemporal FC was reduced in AD+CeVD compared to controls for the lParaHC seed while aMCI subjects showed increases in frontotemporal FC and aMCI+CeVD subjects showed increased in temporoparietal FC for the rParaHC seed. AD and AD+CeVD subjects did not show any increases in inter-network FC for the lParaHC and rParaHC seeds. On the other hand, AD+CeVD subjects showed increases in local medial prefrontal-frontal FC for the mPFC seed and local parietal FC for the PCC and PCUN seeds when compared to healthy controls. Frontal FC was reduced in aMCI subjects for the mPFC seed. Additionally, both AD and AD+CeVD subjects showed reductions in frontoparietal FC for the PCC and PCUN seeds. For the PCUN seed, AD+CeVD subjects also showed increases in frontoparietal FC (Table S2C).

**2.2 Group differences in network FC between CeVD and non-CeVD groups**

Specifically, intra-DMN FC showed decreases in temporoparietal FC in CeVD compared to non-CeVD subjects for the lParaHC, rParaHC, PCC and PCUN seeds with some decreases in medial frontal to parietal FC in aMCI+CeVD compared to aMCI for the PCUN seed. On the other hand, increases in intra-DMN local temporal and parietal FC were observed in the CeVD compared to non-CeVD groups for the lParaHC, PCC and PCUN seeds (Table S3A). Intra-DMN FC was increased in local temporal and parietal regions in CeVD vs. non-CeVD groups. Additionally, intra-ECN frontal FC was increased in aMCI+CeVD compared to aMCI for the rDLPFC seed while frontoparietal FC was increased in AD+CeVD compared to AD subjects for the lPPC seed (Table S3B). No intra-network alterations were observed between CeVD and non-CeVD groups for the mPFC, lDLPFC and rPPC seeds. Furthermore, we observed that inter-network temperoparietal FC was lower in AD+CeVD compared to AD subjects while frontotemporal FC was lower in aMCI+CeVD compared to aMCI subjects for the lParaHC. Similarly, for the rParaHC seed, temporoparietal FC was lower in AD+CeVD compared to AD subjects. However, for the rParaHC, frontotemporal FC was lower in aMCI+CeVD compared to aMCI subjects but higher in AD+CeVD compared to AD subjects. For the PCC seed, inter-network frontoparietal FC was higher in AD+CeVD compared to AD, while parietal FC was higher in aMCI+CeVD compared to aMCI subjects. For the PCUN seed, we observed lower inter-network frontoparietal FC in aMCI+CeVD compared to aMCI but higher frontoparietal FC in AD+CeVD compared to AD subjects. No inter-network alterations were observed for the mPFC seed (Table S3C).

**2.3 Functional connectivity associations with cognition after controlling for the effect of diagnosis and outliers**

Intra-ECN FC was predominantly associated with cognitive disruptions in CeVD subjects while intra-DMN FC was predominantly associated with cognitive disruptions in non-CeVD subjects (Table S7).

**2.4 Structural dysconnectivity is associated with memory and executive functioning deficits in subjects with and without CeVD**

Higher intra-DMN frontoparietal rParaHC-PCC SC was associated with better executive function in subjects with CeVD. Higher intra-ECN frontoparietal rDLPFC-lPPC SC was associated with better executive and attention function in subjects without CeVD. Higher parietal lPPC-rPPC SC was associated with better visual and verbal memory. In subjects with CeVD better frontal lDLPFC-rDLPFC SC was associated with better attention and visual memory. Frontoparietal lDLPFC-rPPC SC was positively associated with executive function and verbal memory. Thus, subjects with and without CeVD showed associations between intra-ECN SC and cognition in both memory and non-memory domains. On the other hand, intra-DMN SC was associated with executive function but not memory function in CeVD subjects (Table S6).

**2.5 Increased executive control network functional connectivity is associated with increased white matter hyperintensity volume in aMCI and AD subjects.**

Higher WMH was associated with higher executive control network functional connectivity including lDLPFC-rMFG FC (rho=0.242; p=0.023) and rDLPFC-rMFG FC (rho=0.293; p=0.006) across all aMCI participants (with and without CeVD, see Figure S5) using Spearman’s correlation between the WMH and FC residuals derived after controlling for age, gender, handedness and ethnicity. This association was also significant for lDLPFC-rMFG (rho=0.263; p=0.010) and rDLPFC-rMFG (rho=0.252; p=0.014) functional connectivity across all AD patients (with and without CeVD) (Figure S5).

**2.6 Higher executive control network functional connectivity is associated with lower executive control network structural connectivity**

We found a significant association between lDLPFC-rDLPFC structural connectivity reduction and lDLPFC-rMFG functional connectivity increase in all AD patients (with and without CeVD, r^2^ = -0.210, p=0.042). We found a similar trend but not significant relationship in the aMCI stage (r^2^ = -0.154; p = 0.15).

**Table S1: Intra-DMN, intra-ECN and inter-network edges**

| **DMN** | | **ECN** | **DMN-ECN** | | |
| --- | --- | --- | --- | --- | --- |
| lParaHC-rParaHC | PCC-mPFC | lDLPFC-rDLPFC | lParaHC-lDLPFC | PCC-lDLPFC | mPFC-lDLPFC |
| lParaHC-PCC | PCUN-mPFC | lDLPFC-lPPC | lParaHC-rDLPFC | PCC-rDLPFC | mPFC-rDLPFC |
| lParaHC-PCUN |  | lDLPFC-rPPC | lParaHC-lPPC | PCC-lPPC | mPFC-lPPC |
| lParaHC-mPFC |  | rDLPFC-lPPC | lParaHC-rPPC | PCC-rPPC | mPFC-rPPC |
| rParaHC-PCC |  | rDLPFC-rPPC | rParaHC-lDLPFC | PCUN-lDLPFC |  |
| rParaHC-PCUN |  | lPCC-rPPC | rParaHC-rDLPFC | PCUN-rDLPFC |  |
| rParaHC-mPFC |  |  | rParaHC-lPPC | PCUN-lPPC |  |
| PCC-PCUN |  |  | rParaHC-rPPC | PCUN-rPPC |  |

Abbreviations: DMN, default mode network; ECN, executive control network; l, left; r, right; DLPFC, dorsolateral prefrontal cortex; PPC, posterior parietal cortex; PCC, posterior cingulate cortex; PCUN, precuneus; ParaHC, parahippocampal cortex.

**Table S2A: Default mode network group differences in functional connectivity**

The “GDS” column represents results from the Geriatric Depression Scale controlled 6mm seed-based functional connectivity analysis on smoothed data. Abbreviations: aMCI, amnestic mild cognitive impairment; AD, Alzheimer’s disease; CeVD, cerebrovascular disease; HC, healthy controls; lParaHC, left parahippocampal cortex; rParaHC, right parahippocampal cortex; mPFC, medial prefrontal cortex; PCC, posterior cingulate cortex; PCUN, precuneus.

**Table S2B: Executive control network group differences in functional connectivity.**

The “GDS” column represents results from the Geriatric Depression Scale controlled 6mm seed-based functional connectivity analysis on smoothed data. Abbreviations: aMCI, amnestic mild cognitive impairment; AD, Alzheimer’s disease; CeVD, cerebrovascular disease; HC, healthy controls; lDLPFC, left dorsolateral prefrontal cortex; rDLPFC, right dorsolateral prefrontal cortex; lPPC, left posterior parietal cortex; rPPC, right posterior parietal cortex.

**Table S2C: Inter-network group differences in functional connectivity.**

The “GDS” column represents results from the Geriatric Depression Scale controlled 6mm seed-based functional connectivity analysis on smoothed data. Abbreviations: aMCI, amnestic mild cognitive impairment; AD, Alzheimer’s disease; CeVD, cerebrovascular disease; HC, healthy controls; lParaHC, left parahippocampal cortex; rParaHC, right parahippocampal cortex; mPFC, medial prefrontal cortex; PCC, posterior cingulate cortex; PCUN, precuneus.

**Table S3A: Intra-DMN group differences in functional connectivity between CeVD and non-CeVD groups.**

The “GDS” column represents results from the Geriatric Depression Scale controlled 6mm seed-based functional connectivity analysis on smoothed data. Abbreviations: aMCI, amnestic mild cognitive impairment; AD, Alzheimer’s disease; CeVD, cerebrovascular disease; lParaHC, left parahippocampal cortex; rParaHC, right parahippocampal cortex; PCC, posterior cingulate cortex; PCUN, precuneus.

**Table S3B: Intra-ECN group differences in functional connectivity between CeVD and non-CeVD groups.**

The “GDS” column represents results from the Geriatric Depression Scale controlled 6mm seed-based functional connectivity analysis on smoothed data. Abbreviations: aMCI, amnestic mild cognitive impairment; AD, Alzheimer’s disease; CeVD, cerebrovascular disease; rDLPFC, right dorsolateral prefrontal cortex; lPPC, left posterior parietal cortex.

**Table S3C: Inter-network group differences in functional connectivity between CeVD and non-CeVD groups.**

The “GDS” column represents results from the Geriatric Depression Scale controlled 6mm seed-based functional connectivity analysis on smoothed data. Abbreviations: aMCI, amnestic mild cognitive impairment; AD, Alzheimer’s disease; CeVD, cerebrovascular disease; lParaHC, left parahippocampal cortex; rParaHC, right parahippocampal cortex; PCC, posterior cingulate cortex; PCUN, precuneus.

**Table S4: Group differences in structural connectivity before and after controlling for total white matter hyperintensity volume.**

Structural connectivity probabilities were logarithmically transformed and normalized for statistical analyses. P-values in green represent comparisons that remained significant after controlling for log transformed white matter hyperintensity volumes. There were no group differences in SC between HCs and aMCI without CeVD subjects. AD+CeVD subjects had the largest reduction in SC at both the intra- and inter-network level compared to both HCs and aMCI with CeVD. We detected a reduction in SC in aMCI+CeVD subjects compared to both controls and aMCI subjects. aMCI+CeVD also showed intra-ECN and inter-network SC reductions compared to AD and aMCI without CeVD subjects respectively. Each cell represents the p-value for significant pair-wise comparisons in SC following correction for multiple comparisons across groups. * refers to comparisons that passed multiple comparisons correction at the threshold of p < 0.05 across all pair-wise comparisons with each ANCOVA model. ** refers to comparisons that passed multiple comparisons correction across the 36 edges.

Abbreviations: HC, Healthy Controls; aMCI, amnestic Mild Cognitive Impairment; CeVD, Cerebrovascular disease; AD, Alzheimer’s disease; DMN, Default Mode Network; ECN, Executive Control Network; lParaHC, left parahippocampus; rParaHC, right parahippocampus; mPFC, medial prefrontal cortex; rPCUN, right precuneus; lPCC, left posterior cingulate cortex; lDLFPC, left dorsolateral prefrontal cortex; rDLFPC, right dorsolateral prefrontal cortex; lPPC, left posterior parietal cortex; rPPC, right posterior parietal cortex; SC, structural connectivity.

**Table S5: Association between functional connectivity and cognitive performance.**

Intra-DMN FC was associated with performance on both memory and non-memory domain primarily in aMCI and AD subjects without CeVD. Temporoparietal intra-DMN FC was associated with memory domain function in subjects with CeVD. Intra-ECN FC was associated with performance on both memory and non-memory domain primarily in aMCI and AD subjects with CeVD. Only frontoparietal intra-ECN FC was associated with memory in subjects without CeVD. All FC-cognitions shown pass the multiple comparisons correction for number of cognitive domains at p<0.0125. Abbreviations: aMCI, amnestic Mild Cognitive Impairment; CeVD, Cerebrovascular disease; AD, Alzheimer’s disease; DMN, Default Mode Network; ECN, Executive Control Network; FC, functional connectivity; lParaHC, left parahippocampus; PCC, posterior cingulate cortex; lDLFPC, left dorsolateral prefrontal cortex; rDLFPC, right dorsolateral prefrontal cortex; lPPC, left posterior parietal cortex; rPPC, right posterior parietal cortex; ANG, angular gyrus; PCUN, precuneus; MCG, middle cingulate gyrus; MFG, middle frontal gyrus; IPG, inferior parietal gyrus; SFG, superior frontal gyrus; l, left; r, right; bil, bilateral.

**Table S6: Association between structural connectivity and cognitive performance.** Intra-DMN SC was positively associated with executive function aMCI and AD subjects with CeVD only. Intra-ECN SC was positively associated with performance on tests of both memory and executive/attention domains in aMCI and AD subjects with and without CeVD. The beta coefficient and the corresponding p-value are stated in each cell for all the significant associations between SC and cognition. All SC-cognitions shown pass the multiple comparisons correction for number of cognitive domains at p<0.0125. Abbreviations: aMCI, amnestic Mild Cognitive Impairment; CeVD, Cerebrovascular disease; AD, Alzheimer’s disease; DMN, Default Mode Network; ECN, Executive Control Network; SC, structural connectivity; rParaHC, right parahippocampus; PCC, posterior cingulate cortex; lDLFPC, left dorsolateral prefrontal cortex; rDLFPC, right dorsolateral prefrontal cortex; lPPC, left posterior parietal cortex; rPPC, right posterior parietal cortex.

**Table S7: Association between functional connectivity and cognitive performance after controlling for diagnosis and removal of outliers.**

Beta coefficients and p-values listed in green represent associations following addition of diagnosis as a covariate to the FC-cognition analyses and those in blue represent associations following the removal of outliers. Intra-DMN FC was associated with performance on both memory and non-memory domain primarily in aMCI and AD subjects without CeVD, especially after the removal of outlier subjects. Temporoparietal intra-DMN FC was associated with memory domain function in subjects with CeVD, after controlling for diagnosis and removal of outlier subjects. Intra-ECN FC was associated with performance on both memory and non-memory domain primarily in aMCI and AD subjects with CeVD. Only frontoparietal intra-ECN FC was associated with memory in subjects without CeVD, after adding diagnosis as well as removal of outlier subjects. * Indicates associations between FC and cognition that passed multiple comparisons correction for number of domains i.e. p<0.0125.

Abbreviations: aMCI, amnestic Mild Cognitive Impairment; CeVD, Cerebrovascular disease; AD, Alzheimer’s disease; DMN, Default Mode Network; ECN, Executive Control Network; FC, functional connectivity; lParaHC, left parahippocampus; PCC, posterior cingulate cortex; lDLFPC, left dorsolateral prefrontal cortex; rDLFPC, right dorsolateral prefrontal cortex; lPPC, left posterior parietal cortex; rPPC, right posterior parietal cortex; ANG, angular gyrus; PCUN, precuneus; MCG, middle cingulate gyrus; MFG, middle frontal gyrus; IPG, inferior parietal gyrus; SFG, superior frontal gyrus; mPFC, medial prefrontal cortex; Amyg, amygdala; l, left; r, right; bil, bilateral.

**Table S8: Region-wise division of cortical infarcts in CeVD groups.**

|  | **Location** | **Frequency** | **Percent** |
| --- | --- | --- | --- |
| **aMCI+CeVD** | None | 13 | 26.0 |
|  | Frontal region | 15 | 30.0 |
|  | Parietal region | 1 | 2.0 |
|  | Temporal region | 1 | 2.0 |
|  | Central region | 9 | 18.0 |
|  | Frontal, Central and Parietal | 3 | 6.0 |
|  | Frontal and Central | 8 | 16.0 |
|  | Total | 50 | 100.0 |
| **AD+CeVD** | None | 27 | 57.4 |
|  | Frontal region | 6 | 12.8 |
|  | Temporal region | 1 | 2.1 |
|  | Frontal and Parietal | 1 | 2.1 |
|  | Central region | 6 | 12.8 |
|  | Frontal, central and parietal | 3 | 6.4 |
|  | Frontal and Central | 3 | 6.4 |
|  | Total | 47 | 100.0 |

Abbreviations: aMCI, amnestic mild cognitive impairment; AD, Alzheimer’s disease; CeVD; cerebrovascular disease.

**Figure S1: Group-averaged functional connectivity maps for each seed for all groups in our study.** Panel A illustrates the group-averaged functional connectivity maps for the left and right DLPFC seeds (left) and left and right PPC seeds (right) for all the 5 groups. Panel B illustrates the group-averaged functional connectivity maps for the PCC, PCUN and mPFC (left) and the left and right ParaHC seeds (right). Maps are thresholded at a height threshold of FWE corrected p<0.001, extent threshold of 1000 voxels for all seeds, except bilateral ParaHC seeds at a height threshold of uncorrected p<0.001.

Abbreviations: HC, healthy controls; aMCI, amnestic mild cognitive impairment; aMCI+CeVD; amnestic mild cognitive impairment with cerebrovascular disease; AD, Alzheimer’s disease; AD+CeVD, Alzheimer’s disease with cerebrovascular disease; l, left; r, right; DLPFC, dorsolateral prefrontal cortex; PPC, posterior parietal cortex; PCC, posterior cingulate cortex; PCUN, precuneus; ParaHC, parahippocampal cortex.

**Figure S2: Representative intra-network and inter-network structural connections obtained from probabilistic tractography.** Intra-DMN, intra-ECN and DMN-ECN white matter connections are shown: between the mPFC and the PCC seeds for intra-DMN; between the lDLPFC and rDLPFC seeds for intra-ECN and between lPPC and PCC seeds for DMN-ECN. These representative images were obtained from one example healthy older adult using DTIStudio software.

Abbreviations: HC, Healthy Controls; DMN, Default Mode Network; ECN, Executive Control Network; mPFC, medial prefrontal cortex; PCC, posterior cingulate cortex; lDLFPC, left dorsolateral prefrontal cortex; rDLFPC, right dorsolateral prefrontal cortex; lPPC, left posterior parietal cortex.


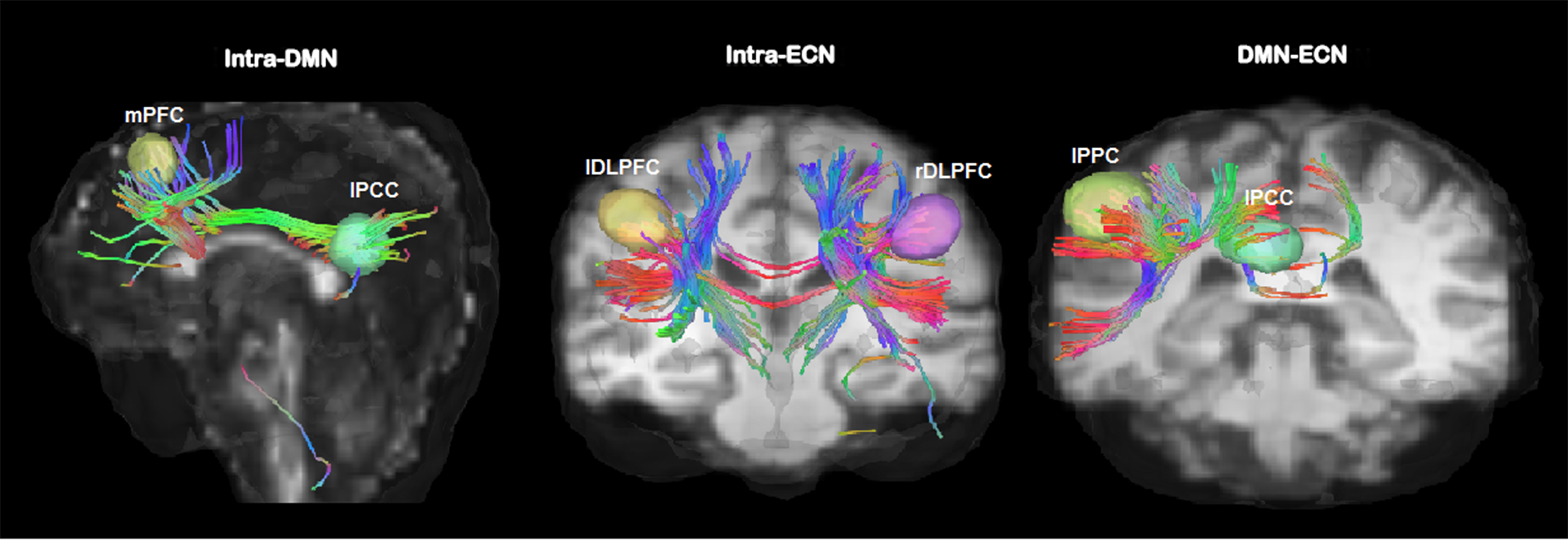


**Figure S3: Default mode network group differences between aMCI and aMCI+CeVD subjects and healthy controls.**

Group functional connectivity difference maps were overlaid on the MNI template brain. The maps highlight regions showing increased (hot colour) patient groups compared to HCs for the DMN. aMCI+CeVD subjects showed increased intra-DMN parietal FC for the PCUN and mPFC seeds.

Abbreviations: HC, Healthy Controls; aMCI, amnestic Mild Cognitive Impairment; CeVD, Cerebrovascular disease; AD, Alzheimer’s disease; FC, functional connectivity; DMN, Default Mode Network; PCUN, precuneus; mPFC, medial prefrontal cortex; PCG, posterior cingulate gyrus; MCG, middle cingulate gyrus; l, left; bil, bilateral.

**Figure S4 – Executive control network group differences between aMCI and aMCI+CeVD subjects and healthy controls.**

Group functional connectivity difference maps were overlaid on the MNI template brain. The maps highlight regions showing increased (hot colour) or decreased functional connectivity (cold colour) in patient groups compared to HCs for the ECN. aMCI+CeVD subjects showed increased intra-ECN inferior frontal FC for the rDLPFC seed and decreased superior and middle frontal FC for the rDLPFC and rPPC seeds

Abbreviations: HC, Healthy Controls; aMCI, amnestic Mild Cognitive Impairment; CeVD, Cerebrovascular disease; AD, Alzheimer’s disease; FC, functional connectivity; ECN, Executive Control Network; rDLPFC, right dorsolateral prefrontal cortex; lPPC, left posterior parietal cortex; SFG, superior frontal gyrus; MFG, middle frontal gyrus; IFG, inferior frontal gyrus; l, left; r, right.

**Figure S5: Increased executive control network functional connectivity is associated with increased white matter hyperintensity volume in aMCI and AD subjects.**

Higher WMH volume was associated with increased intra-ECN functional connectivity between rDLPFC-rMFG was associated across all aMCI participants (aMCI + aMCI+CeVD) subjects and between lDLPFC-rMFG across all AD participants (AD + AD+CeVD). Spearman’s rho and p-values are reported.

Abbreviations: aMCI, amnestic mild cognitive impairment; AD, Alzheimer’s disease; CeVD, cerebrovascular disease; ECN, executive control network; WMH, white matter hyperintensities; r, right; l, left; DLPFC, dorsolateral prefrontal cortex; MFG, middle frontal gyrus.

Supplementary References

1. Hilal S, Chai YL, Ikram MK, Elangovan S, Yeow TB, Xin X, Chong JY, Venketasubramanian N, Richards AM, Chong JP, et al: **Markers of cardiac dysfunction in cognitive impairment and dementia.** *Medicine (Baltimore)* 2015, **94:**e297.

2. Hilal S, Tan CS, Xin S, Amin SM, Wong TY, Chen C, Venketasubramanian N, Ikram MK: **Prevalence of cognitive impairment and dementia in Malays - Epidemiology of Dementia in Singapore Study.** *Curr Alzheimer Res* 2015.

3. Ong YT, Hilal S, Cheung CY, Venketasubramanian N, Niessen WJ, Vrooman H, Anuar AR, Chew M, Chen C, Wong TY, Ikram MK: **Retinal neurodegeneration on optical coherence tomography and cerebral atrophy.** *Neurosci Lett* 2015, **584:**12-16.
